# Characterization of a Pyroptosis-Related Signature for Prognosis Prediction and Immune Microenvironment Infiltration in Prostate Cancer

**DOI:** 10.1155/2022/8233840

**Published:** 2022-04-27

**Authors:** Guian Zhang, Yong Luo, Weimin Dong, Weide Zhong

**Affiliations:** ^1^School of Medicine, South China University of Technology, Guangzhou, China; ^2^Department of Urology, Guangdong Key Laboratory of Clinical Molecular Medicine and Diagnostics, Guangzhou First People's Hospital, School of Medicine, South China University of Technology, Guangzhou, China; ^3^Department of Urology, The Second People's Hospital of Foshan, Affiliated Foshan Hospital of Southern Medical University, Foshan 528000, China; ^4^Department of Urology, The Fifth Affiliated Hospital of Guangzhou Medical University, Guangzhou, China

## Abstract

This study was aimed at constructing a pyroptosis-related signature for prostate cancer (PCa) and elucidating the prognosis and immune landscape and the sensitivity of immune checkpoint blockade (ICB) therapy in signature-define subgroups of PCa. We identified 22 differentially expressed pyroptosis-related genes in PCa from The Cancer Genome Atlas (TCGA) database. The pyroptosis-related genes could divide PCa patients into two clusters with differences in survival. Seven genes were determined to construct a signature that was confirmed by qRT-PCR to be closely associated with the biological characteristics of malignant PCa. The signature could effectively and independently predict the biochemical recurrence (BCR) of PCa, which was validated in the GSE116918 and GSE21034. We found that patients in the high-risk group were more prone to BCR and closely associated with high-grade and advanced-stage disease progression. Outperforming clinical characteristics and nine published articles, our signature demonstrated excellent predictive performance. The patients in the low-risk group were strongly related to the high infiltration of various immune cells including CD8+ T cells and plasma B cells. Furthermore, the high-risk group with higher TMB levels and expression of immune checkpoints was more likely to benefit from immune checkpoint therapy such as PD-1 and CTLA-4 inhibitors. The sensitivity to chemotherapy, endocrine, and targeted therapy showed significant differences in the two risk groups. Our signature was a novel therapeutic strategy to distinguish the prognosis and guide treatment strategies.

## 1. Introduction

Prostate cancer (PCa) is the second most widespread male cancer with high lethality, causing more than 370000 deaths worldwide in 2020 [1]. Meanwhile, more than one-third of patients eventually experience biochemical recurrence (BCR) after definitive treatment [2]. Patients with BCR were more likely to develop clinical recurrence, metastases, and cancer-specific mortality [3]. Therefore, early detection of BCR was essential for the management and treatment of PCa patients. The existing clinical indicators cannot effectively predict BCR and guide treatment, necessitating representative and robust clinical models to promote preclinical translational and mechanistic studies of treatment in PCa.

Pyroptosis is considered to be a form of programmed cell necrosis triggered by proinflammatory signals and associated with inflammation [4]. Pyroptotic cells undergo cytoplasmic swelling and membrane pore formation, leading to loss of plasma membrane integrity and ultimately to leakage of cytoplasmic contents. The occurrence of pyroptosis requires the activation of caspase-1, which is responsible for the maturation of proinflammatory cytokines through inflammasome-dependent pathways, such as interleukin 1 *β* (IL-1*β*) and IL-18 [5]. Meanwhile, gasdermin D (GSDMD) cleaved by activated caspase-1 locks into the plasma membrane to form pores [6]. More and more studies on the relationship between pyroptosis and tumors had shown that pyroptosis played an important role in the proliferation, invasion, and metastasis of tumor cells and affected the prognosis and therapeutic effects of tumors. GSDME-mediated pyroptosis promoted the development of colitis-related colorectal cancer, inducing tumor cell proliferation and proliferating cell nuclear antigen expression [7]. Gasdermin E-dependent pyroptosis might be indispensable in mediating the immunotherapy response of BRAF mutant melanoma [8].

The tumor microenvironment (TME) has been confirmed to play a central role in tumorigenesis, immune escape, progression, and metastasis [9]. Tumor cells actively secrete inflammatory factors and growth factors to recruit stromal cells, inflammatory, and immune cells. The interaction between tumor cells and nontumor cells shapes TME, which in turn affects tumor progression and evades immune surveillance [10]. Characterized as inflammatory, pyroptosis recruited and activated immune cells through the inflammatory factors released during cell death to bridge innate immunity and adaptive immunity to regulate the TME and induce immune responses [11]. Meanwhile, neoantigens produced during the process of pyroptosis further induced new immune responses and hindered the development of tumors [12]. The study by Z. Zhang et al. showed that the infiltration of CD8+ T cells and natural killing cells in the pyroptosis-activated TME could promote pyroptosis and form a positive feedback loop [13]. The important role of pyroptosis in the efficacy of cancer immunotherapy, such as immune checkpoint blockade (ICB), and the new approaches of pyroptosis to aid immunotherapy were receiving increasing attention [14]. Therefore, there was a need to identify the different risk stratification of PCa patients for immunotherapy through a comprehensive and deep insight into TME by pyroptosis.

In this study, we sought to develop a prognostic signature for PCa, which can effectively stratify patients and predict the prognosis and treatment efficacy of patients with different risk levels. The results revealed that the predictive ability of our signature was superior to traditional clinical features. On this basis, we systematically explored the role of the signature in the TME. Our signature was a promising prognostic biomarker to guide and determine the subgroup of PCa patients more suitable for endocrine therapy, chemotherapy, and immunotherapy.

## 2. Materials and Methods

### 2.1. Data Source and Preprocessing

Transcriptome RNA sequencing data and corresponding clinical information of PCa samples, which was the training cohort, were downloaded from the TCGA program (https://tcga-data.nci.nih.gov/tcga/). The GSE116918 dataset as testing cohort and GSE21034 dataset as validation cohort were extracted from the Gene Expression Omnibus (GEO) dataset (https://www.ncbi.nlm.nih.gov/geo/). The ComBat algorithm of SVA package was applied to correct the batch impact of nonbiotechnical bias. The training cohort was appointed to build signature, and the testing and validation cohorts were used to validate it. The R package *maftools* was used to visualize the mutation landscape, and the CNV feature in human chromosomes was investigated by the *Rcircos* package. The *rms* package was used to build a predictive nomogram for predicting the 1-, 2-, and 3-year overall survival.

### 2.2. Identification of Differentially Expressed Pyroptosis-Related Genes

A total of 33 pyroptosis-related genes were selected based on the previously published literature [15]. The difference in pyroptosis-related genes with a *P* value < 0.05 was identified by *limma* package. We constructed a protein-protein interaction (PPI) network using the Search Tool for Retrieval of Interacting Genes (STRING).

### 2.3. Consensus Clustering

To identify different pyroptosis modifications, we applied consensus clustering to identify different pyroptosis patterns associated with the expression of pyroptosis-related genes. The *ConsensuClusterPlus* package was applied to determine the number of clusters and their stability, performing 1000 replications. The clusters were selected based on the relative change in the area under the cumulative distribution function (CDF) curve, the number of samples in the cluster, and the relevance of the cluster.

### 2.4. Construction of the Signature

The Cox regression analysis was conducted to assess the correlation between the expression level of each gene and its prognosis. Furthermore, we obtained candidate gene through the least absolute shrinkage and selection operator (Lasso) with 10-fold cross-validation. In the end, we kept the 7 genes and the coefficients, and the penalty parameter (*λ*) was determined by the minimum criterion. The formula to calculate the risk score was as follows: Risk Score = ∑_*i*_^*λ*^*βiSi*, where *β* is the coefficients and *S* is the gene expression level.

### 2.5. Evaluation of the Signature

The area under curve (AUC) value of ROC curves was used to assess the sensitivity and specificity. A risk score was assigned to each patient according to the signature. Furthermore, we divided the PCa patients into high- and low-risk groups by the median value of risk score. Survival curves were plotted by the Kaplan-Meier analysis to assess the overall survival of patients in the high- and low-risk groups. The univariate and multivariate Cox regression analyses were implemented to evaluate the independent prognostic value. These R software packages include *timeROC*, *survival*, and *survminer*.

### 2.6. Functional Enrichment Analysis

Gene Ontology (GO) including biological process (BP), cellular component (CC), and molecular function (MF) categories and the Kyoto Encyclopedia of Genes and Genomes (KEGG) were analyzed by the *clusterProfiler* R package.

### 2.7. Immune Landscape and TIDE Analysis

In order to explore the difference of the abundance of immune infiltrates in the high- and low-risk groups, we used the algorithms including EPIC, XCELL, MCPCOUNTER, QUANTISEQ, CIBERSORT-ABS, CIBERSORT, and TIMER to score the infiltration of each immune cell subtype. The significance threshold was set to a *P* value less than 0.05. The Wilcoxon sign-rank test was used to analyze the difference in the abundance of immune infiltrating cells between the high- and low-risk groups. The tumor immune dysfunction and exclusion (TIDE) of the PCa patients was calculated from the website (http://tide.dfci.harvard.edu/). The tumor inflammation signature (TIS) score was computed as the mean of log2-scale normalized expression of 18 signature genes [16].

### 2.8. Association between the Signature and the Treatments

To investigate the potential role of the signature in immunotherapy, we analyzed the relationship between the signature and immune checkpoints expression. Here, we adopted the *ggpubr* package. In addition, we explored the function of signature in endocrine therapy and chemotherapy by analyzing the half-maximal inhibitory concentration (IC50) of the drugs. The difference in targeted therapy between the high- and low-risk groups was found by the Wilcoxon signed-rank test. The R packages used here were *pRRophetic* and *ggplot2.* NCI-60 database of 60 different tumor cell lines from 9 different tumor types was provided by CellMiner (https://discover.nci.nih.gov/cellminer). Pearson's correlation analysis was carried to analyze the drug sensitivity between the expression of genes and 263 drugs approved by the FDA or in clinical trials.

### 2.9. Cell Line Culture and qRT-PCR

All human cell lines were purchased from the American Type Culture Collection (ATCC, USA), including DU145, PC3, and BPH-1. All cells were cultured in Roswell Park Memorial Institute (RPMI) 1640 medium (Gibco, USA; catalog number: C11875500BT) supplemented with 10% fetal bovine serum (FBS; Gibco, USA; Cat.10270–106), 0.1 mg/mL streptomycin, and 100 U/mL penicillin (Gibco, USA; catalog number: 15,140–122) and were maintained in a humidified incubator at 37°C containing 5% CO_2_. Total RNA was obtained with the RNeasy mini kit (QIAGEN, Germany, Cat. No. 74,104) and reverse transcribed with the RT kit (TaKaRa, Japan, Cat. No. NR037A). The cDNA products were then subjected to real-time PCR using Fast SYBR® Green Master Mix (Life technology, USA; Cat. No: 4,385,610). The sequences of all primers used for PCR were documented in the supplementary materials.

### 2.10. Statistical Analysis

All statistical analyses were applied by R version 4.1.1 (Institute for Statistics and Mathematics, Vienna, Austria; https://www.r-project.org), and some related packages were applied to all statistical analyses. *P* < 0.05 was considered the significantly statistical difference.

## 3. Result

### 3.1. Screening Differentially Expressed Pyroptosis-Related Genes

**Figure 1:**
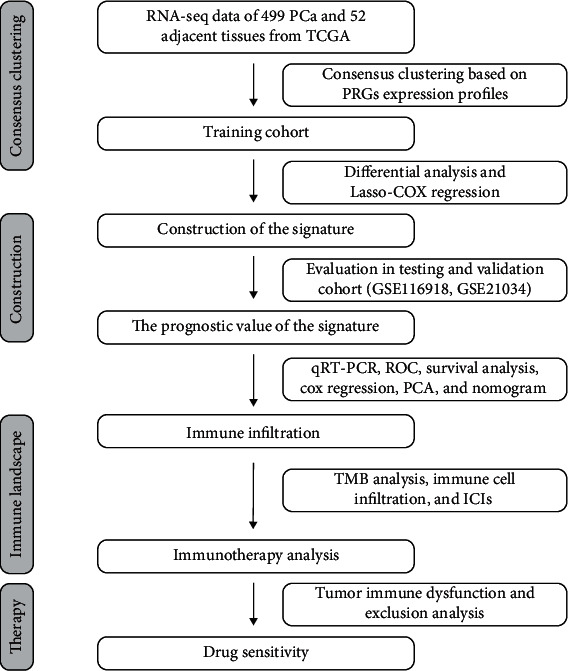
The workflow of this study.

**Figure 2:**
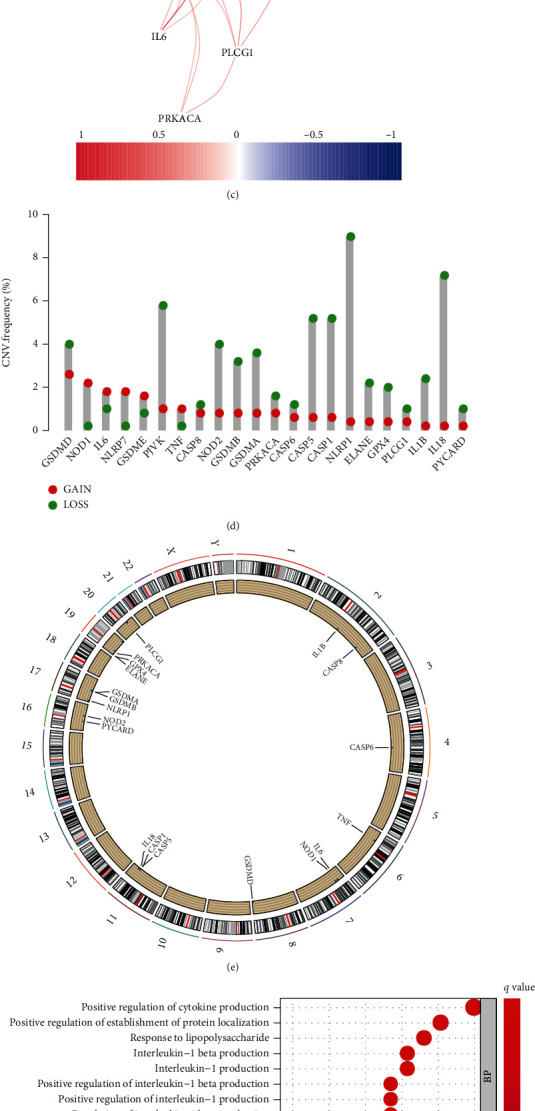
Expression and interactions of the pyroptosis-related genes in PCa. (a) Heatmap of differentially expressed pyroptosis-related genes in tumor and normal tissues. (b) Protein-protein interaction network of 22 DEGs. (c) The correlation network of DEGs. (d) The CNV variation frequency of DEGs. (e) The location of CNV alteration of DEGs on chromosomes. (f) Bubble graph for GO enrichment and (g) KEGG pathways.

The brief process of this research was depicted in Figure 1. Initially, we compared the expression of 33 pyroptosis-related genes in 52 normal tissues and 499 PCa samples from the TCGA database and identified 22 differentially expressed genes (DEGs), which were depicted in the heatmap (all *P* < 0.001) (Figure 2(a)). Protein-protein interaction (PPI) analysis with the minimum required interaction score of 0.9 was employed to investigate the interactions of these DEGs. CASP1, CASP8, IL1B, and PYCARD were identified as hub genes (Figure 2(b)). Furthermore, the correlation network of the DEGs was illustrated in Figure 2(c). The analysis of CNV alteration frequency exhibited that most DEGs were focused on copy number reduction (Figure 2(d)). We further annotated the sites of CNV alterations of DEGs on the chromosome (Figure 2(e)). In order to further explore the biological processes and potential molecular mechanisms that the DEGs involved, we conducted GO analysis and KEGG pathway, revealing the participation of many biological processes and signaling pathways (Figures 2(f) and 2(g)).

### 3.2. Classification of PCa Patients Based on Pyroptosis-Related Genes

**Figure 3:**
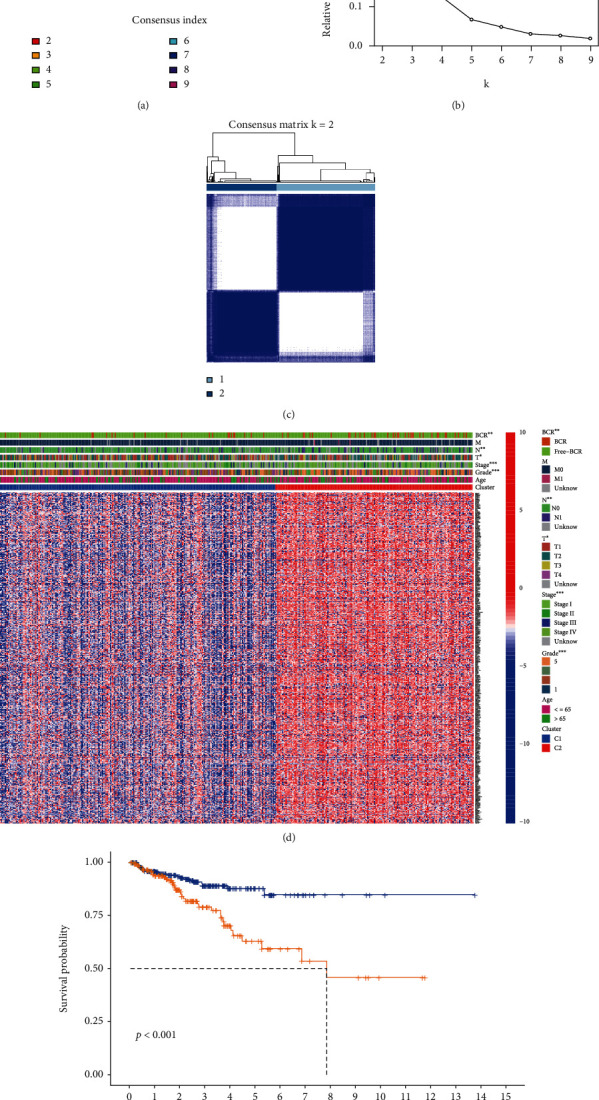
Clinical characteristics of PCa clusters. (a) CDF curves in clustering PCa patients. (b) Relative changes in the AUC of CDF curves. (c) PCa patients were divided into two clusters based on consensus clustering matrix. (d) The clinical characteristics of the two clusters in the heatmap. (e) Survival analysis in the two clusters.

The empirical CDF was depicted to identify the optimum *k* values for the distribution of samples with maximal stability (Figures 3(a) and 3(b)). The result of consensus matrices suggested that PCa patients can be divided into two completely different clusters when clustering variable (*k*) = 2 (Figure 3(c)). We found significant differences in the clinical characteristics including BCR, M stage, N stage, T stage, tumor stage, and tumor grade between these two different clusters (Figure 3(d)). In addition, the Kaplan-Meier survival analysis confirmed that patients in cluster 2 had a shorter BCR-free time than those in cluster 1 (*P* < 0.001) (Figure 3(e)).

### 3.3. Construction and Evaluation of Prognostic Signature for PCa

**Figure 4:**
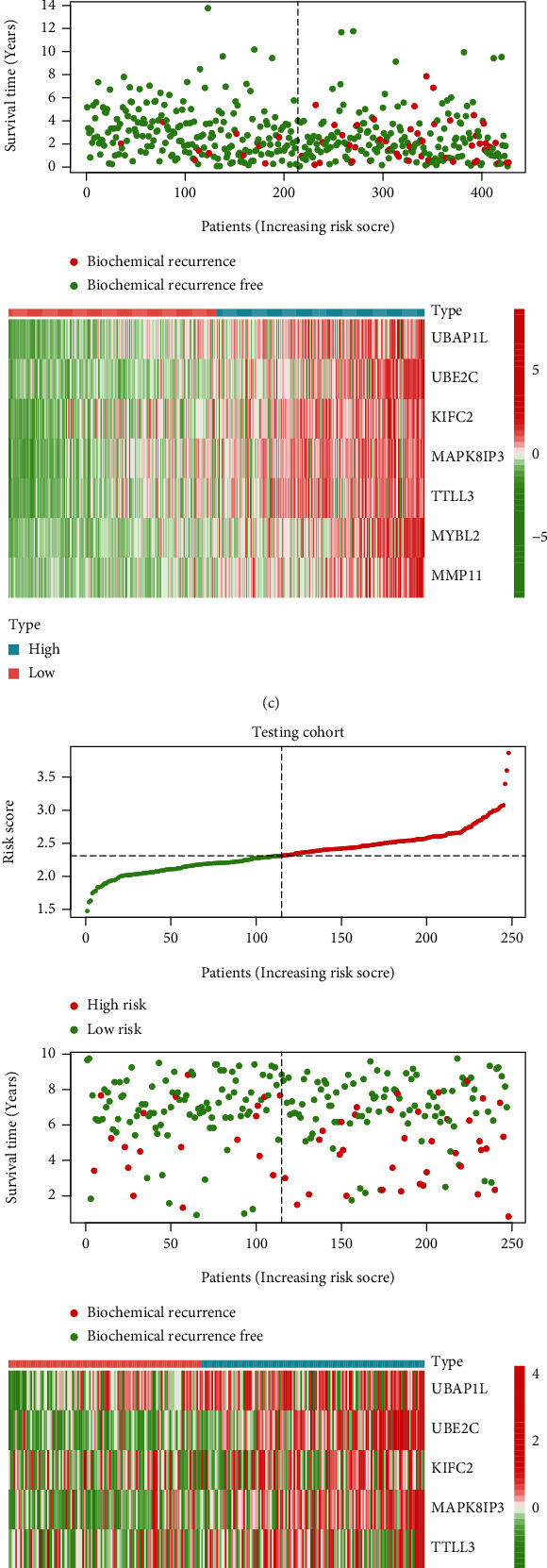
Identification of a signature to predict the BCR of PCa. (a and b) Process of variable selection in Lasso Cox regression and the optimal values of the penalty parameter were determined by 10-fold cross-validation in the training cohort. The risk score, survival status, and heatmap of the signature in the (c) training cohort, (d) testing cohort, and (e) validation cohort.

**Figure 5:**
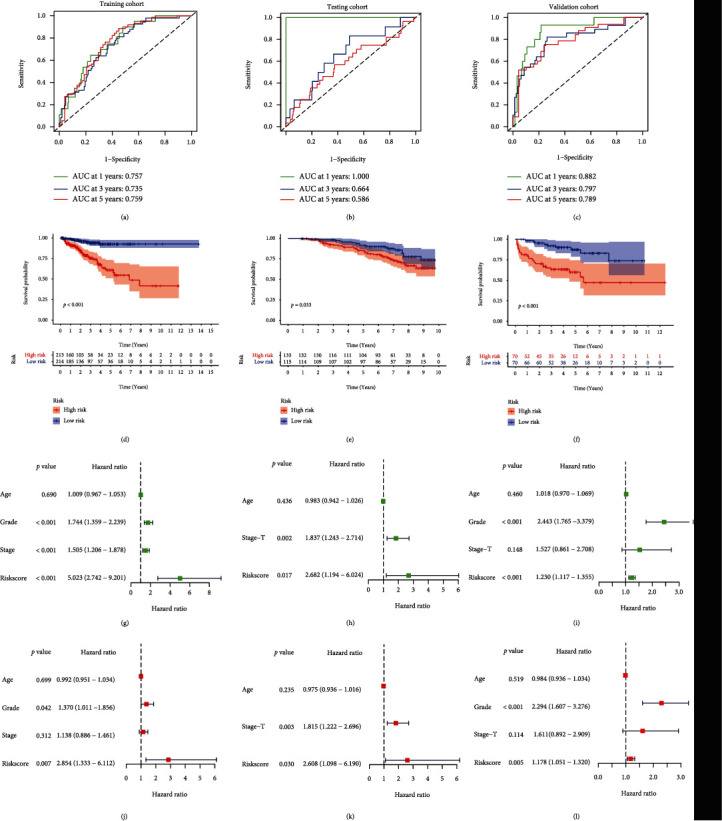
Validation of the signature in multiple cohorts. Time-dependent ROC curves analysis in the (a) training cohort, (b) testing cohort, and (c) validation cohort. The Kaplan-Meier survival curves based on the signature in the (d) training cohort, (e) testing cohort, and (f) validation cohort. Univariate analysis in the (g) training cohort, (h) testing cohort, and (i) validation cohort. Multivariate Cox regression in the (j) training cohort, (k) testing cohort, and (l) validation cohort.

To identify a specific prognostic signature for disease diagnosis and treatment, we explored differentially expressed genes between the above two clusters. Then, we performed univariate Cox regression and Lasso regression analysis, in which the best values of the penalty parameter were determined by 10-fold cross-validation (Figures 4(a) and 4(b)). Finally, 7 effective genes for the construction of the risk signature were determined. The PCa patients were stratified into high-risk and low-risk groups according to the median risk score as the cut-off point. The distribution of risk score showed a significant difference in BCR-free time among the training cohort, testing cohort, and independent external validation cohort, with a gradual increase in the probability of BCR as the risk score increased (Figures 4(c) – 4(e)). Furthermore, we performed time-dependent ROC analysis and calculated the AUC at 1, 3, and 5 years, showing good sensitivity and specificity of the signature for prognosis of PCa patients in three cohorts (Figures 5(a) – 5(c)). The result of the Kaplan-Meier survival curve indicated that the patients in the high-risk group suffered shorter BCR-free time, showing the same outcome in all three cohorts (Figures 5(d) – 5(f)). The univariate and multivariate Cox regression proved that the signature could serve as a robust and independent prognostic factor for PCa patients (Figures 5(g) – 5(l)).

### 3.4. Distribution Patterns of the High-Risk and Low-Risk Groups

**Figure 6:**
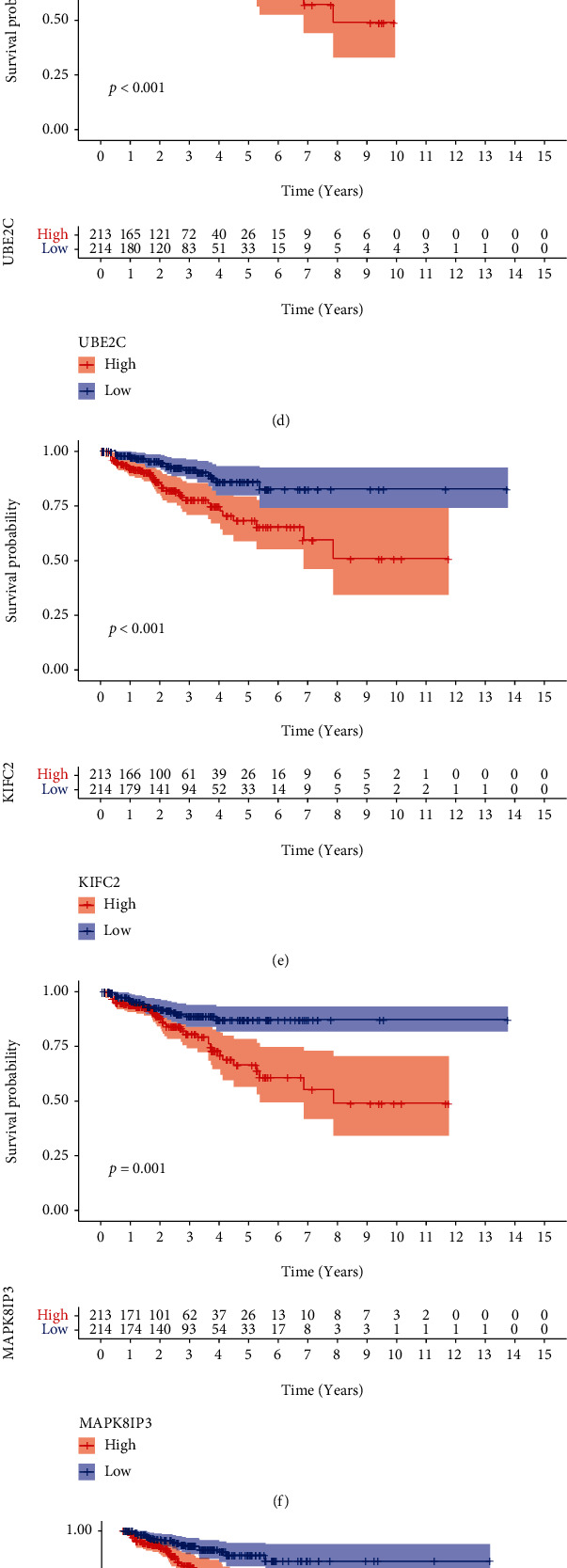
Distribution pattern and Kaplan-Meier survival analysis. (a) 2D PCA plot and t-SNE analysis between the high- and low-risk groups in the training cohort. (b) 2D PCA plot and t-SNE analysis between the two groups in the testing cohort. (c–i) The Kaplan-Meier survival curve of 7 genes between the two groups.

**Figure 7:**
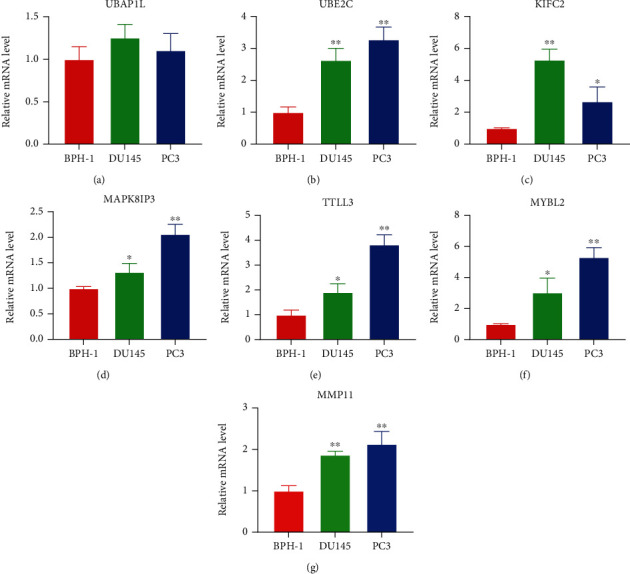
The expression of seven genes in PCa cell lines. (a–g) The relative mRNA levels of UBAP1L, UBE2C, KIFC2, MAPK8IP3, TTLL3, MYBL2, and MMP11 in DU145, PC3, and BPH-1.

PCA and t-SNE analyses were conducted to reduce dimensionality and showed a satisfactory separation between the high- and low-risk groups. The distribution of the high- and low-risk groups tended to be in different directions (Figures 6(a) and 6(b)). Furthermore, we explored the impact of the 7 genes used to construct the signature on BCR-free time. Surprisingly, patients had higher probability of BCR when each of these genes was highly expressed (Figures 6(c) – 6(d)). We further analyzed the mRNA expression of the 7 genes used to construct the signature in two PCa cell lines (DU145 and PC3) and benign prostatic hyperplasia cell (BPH-1) by qRT-PCR assays. These results indicated that the expression levels of UBE2C, KIFC2, MAPK8IP3, TTLL3, MYBL2, and MMP11 were significantly upregulated in PCa cell lines, except for UBAP1L which did not show significant differences (Figures 7(a) – 7(g)).

### 3.5. Correlation between Clinicopathological Characteristics and the Signature

**Figure 8:**
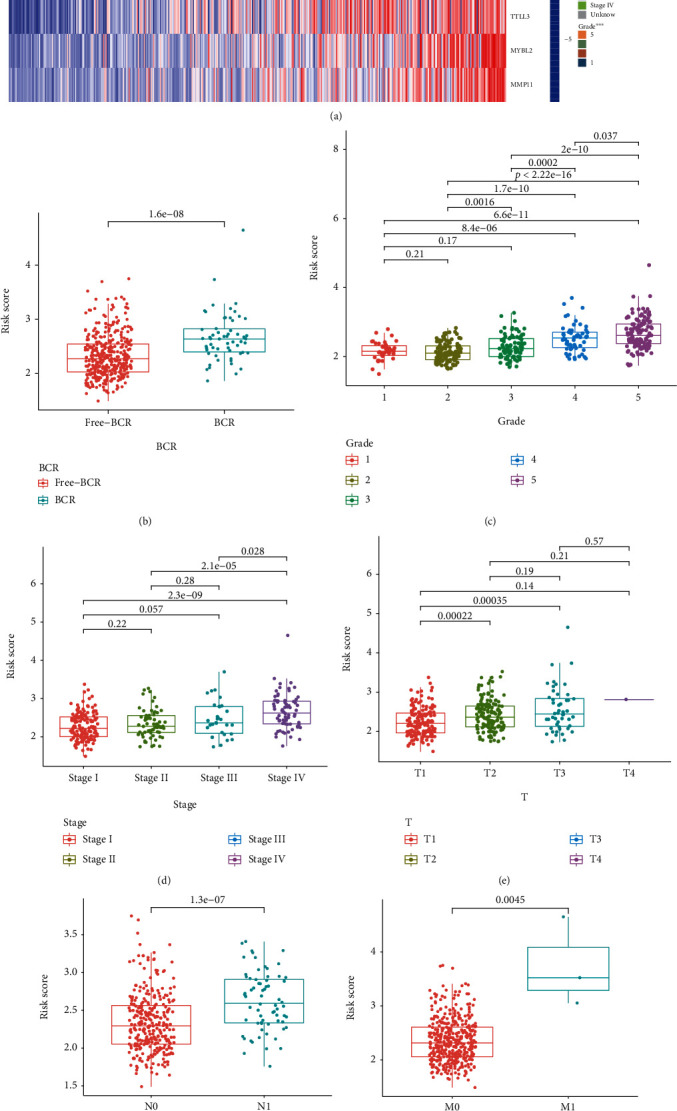
Evaluating the relationship between the signature and clinical characteristics of PCa. (a) The distribution of clinicopathological factors between the high- and low-risk groups. Risk scores were significantly associated with BCR (b), tumor grade (c), tumor stage (d), T stage (e), N stage (f), and M stage (g).

**Figure 9:**
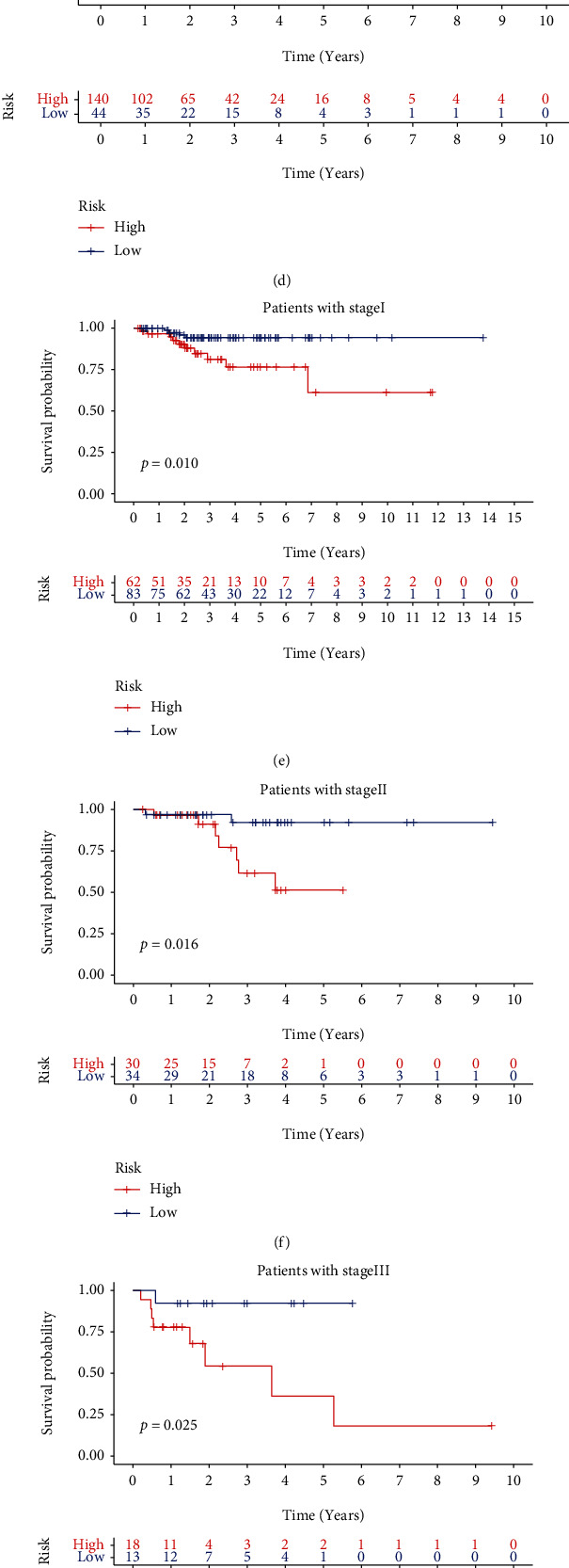
Stratification survival analyses. (a–j) The Kaplan-Meier curve analyses of overall survival in subgroups stratified by different clinical features.

The distributed patterns between the signature and clinicopathological characteristics were illustrated on the heatmap (Figure 8(a)). The BCR, M stage, N stage, T stage, tumor stage, tumor grade, and age were diversely distributed in the high- and low-risk groups. To further investigate whether the signature was closely related to different clinicopathological conditions, we found that the clinical features including BCR, tumor grade, tumor stage, T stage, N stage, and M stage were significantly associated with the signature (Figures 8(b) – 8(g)). The high-grade and advanced-stage patients were more likely to be related to the high-risk group. In addition, the low-risk group was more inclined to low grade and early stage, which were equivalent to a better prognosis. We further divided PCa patients into different stratified groups according to age, gender, tumor grade, tumor stage, and T stage. There were significant differences between the high- and low-risk groups, suggesting that the low-risk group had longer BCR-free time in all stratification subgroups. (Figures 9(a) – 9(k)) Therefore, the signature might be significantly associated with the progression of PCa and had broad applicability and feasibility for prognosis prediction.

### 3.6. Construction and Evaluation of the Nomogram

**Figure 10:**
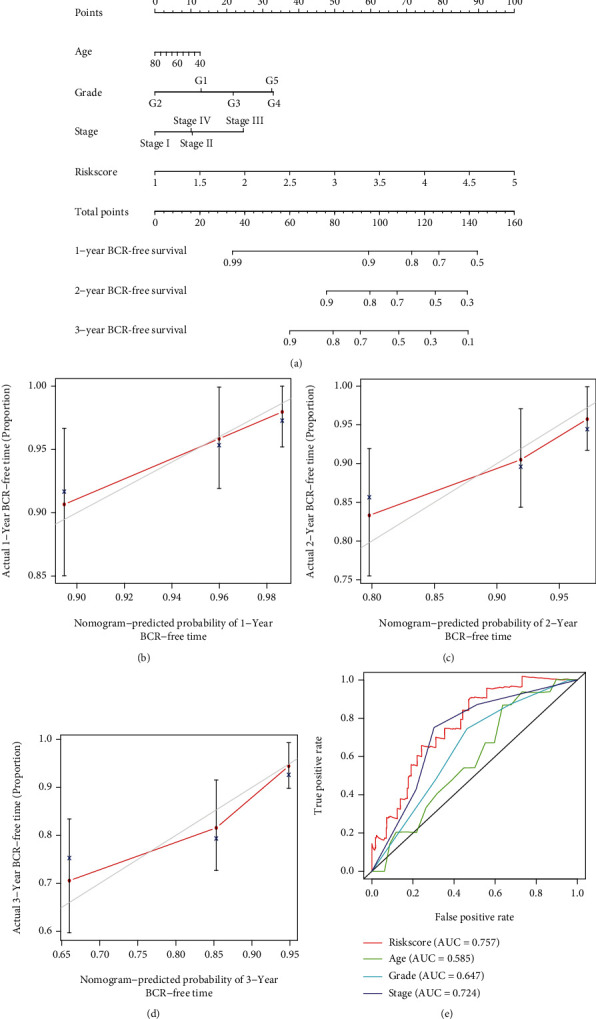
Construction and validation of nomogram. (a) The nomogram for predicting the probability of the 1-, 2-, and 3-year BCR-free survival. (b–d) Calibration curves for the validation of the nomogram. (e) Time-dependent ROC curves analysis of signature and the clinical factors.

We constructed a nomogram containing risk scores and clinical characteristics to predict the 1-, 2-, and 3-year BCR probability of PCa patients. A higher total score in the nomogram represented a worse prognosis (Figure 10(a)). The calibration chart displayed excellent agreement between observed and predicted rates at 1, 2, and 3 years (Figures 10(b) – 10(d)). By comparing the AUC between the signature and clinical features, we found that our signature can predict BCR more accurately (Figure 10(e)). Thus, our nomogram based on the signature had good predictive ability in clinical practice.

### 3.7. Comparison with Other Gene Expression Signatures

**Figure 11:**
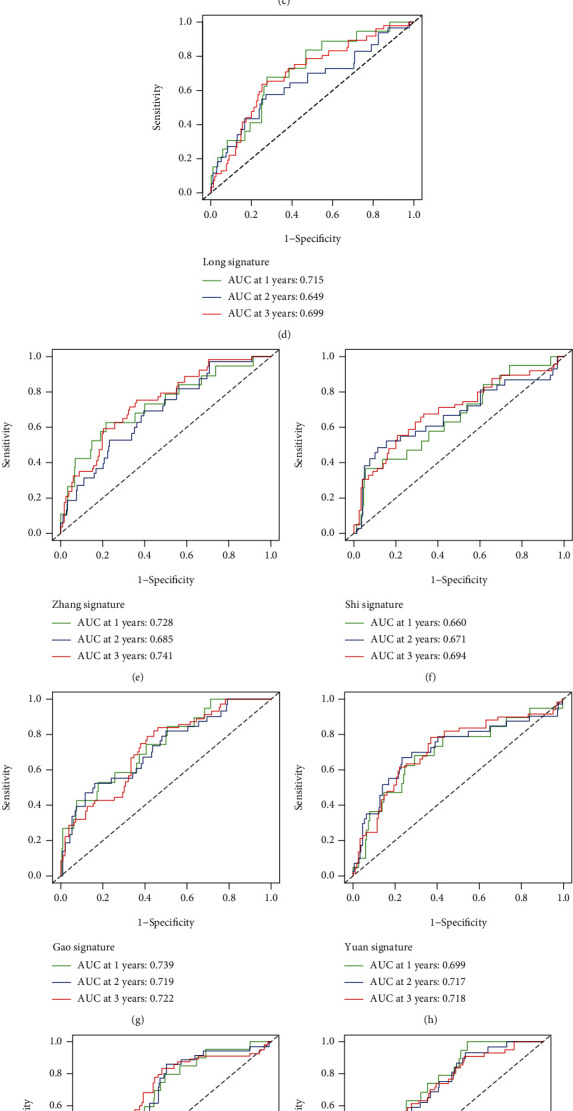
Comparison with other 9 published gene signatures (a–j). (k) C-index of signatures.

To determine whether our signature was superior to other signatures, we compared the signatures constructed for PCa in 9 published articles [17–25]. We found that the accuracy and stability of our signature in 1, 2, and 3 years were better than those of the nine signatures in the ROC curves analysis (Figures 11(a) – 11(j)). Then, in order to further compare our signature with the predicted performance of these signatures, we calculated the concordance index (C-index). As the results depicted, the C-index of our signature was 0.731 (Figure 11(k)), which was better than other signatures.

### 3.8. Landscape of Somatic Mutations in PCa

**Figure 12:**
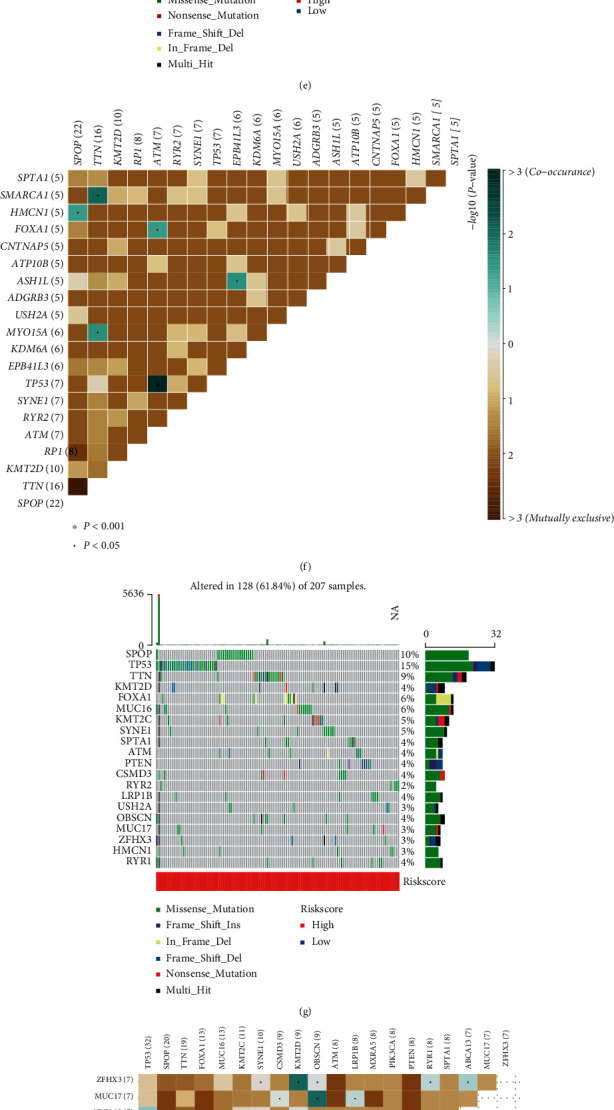
TMB analyses between the high- and low-risk groups. (a) TMB levels in the high- and low-risk groups. (b) The relationship between TMB levels and the risk score. (c) The Kaplan-Meier survival curves of patients with high and low TMB levels. (d) The Kaplan-Meier survival curves of patients with different TMB levels and risk groups. (e–j) Detailed mutation information in the two groups.

We analyzed the TMB level of the high- and low-risk groups and found that the TMB level of the high-risk group was higher than the TBM level of the low-risk group and was proportional to the risk score (Figures 12(a) and 12(b)). PCa patients with high TMB levels were more likely to develop BCR (Figure 12(c)). After further dividing the patients into the high- and low-risk groups by TMB level, we noticed that the patients in the high-risk group with high TMB levels had the shortest BCR-free time (Figure 12(d)). We then compared the 20 genes with the highest mutation frequencies in the high- and low-risk groups, showing that these genes were mutated more frequently in the high-risk group, with more significant gene-to-gene coincidence and exclusivity relationships (Figures 12(e) – 12(j)).

### 3.9. Evaluation the Immune Landscape of PCa

**Figure 13:**
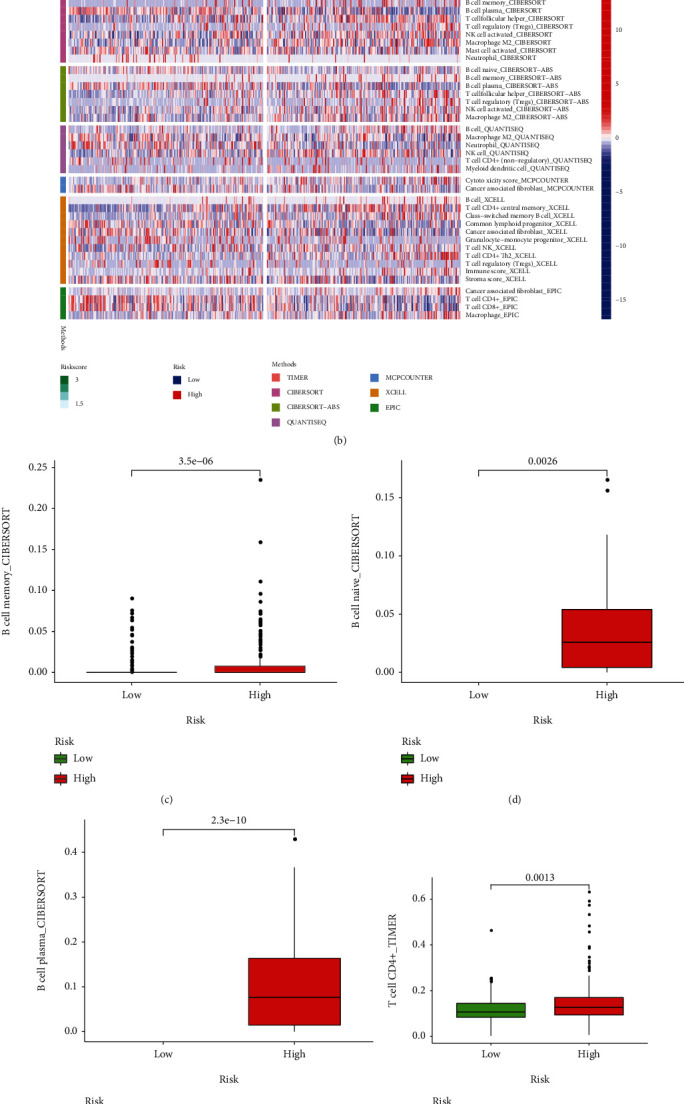
CCorrelation between the signature and the immune infiltration. (a) The difference between the signature and tumor-infiltrating immune cells in multiple algorithms. (b) The distribution of the immune cells in the high- and low-risk groups. The abundance of (c) B memory cells, (d) B naive cells, (e) plasma B cells, (f) CD4+ T cells, and (g) CD8+ T cells in the two groups.

**Figure 14:**
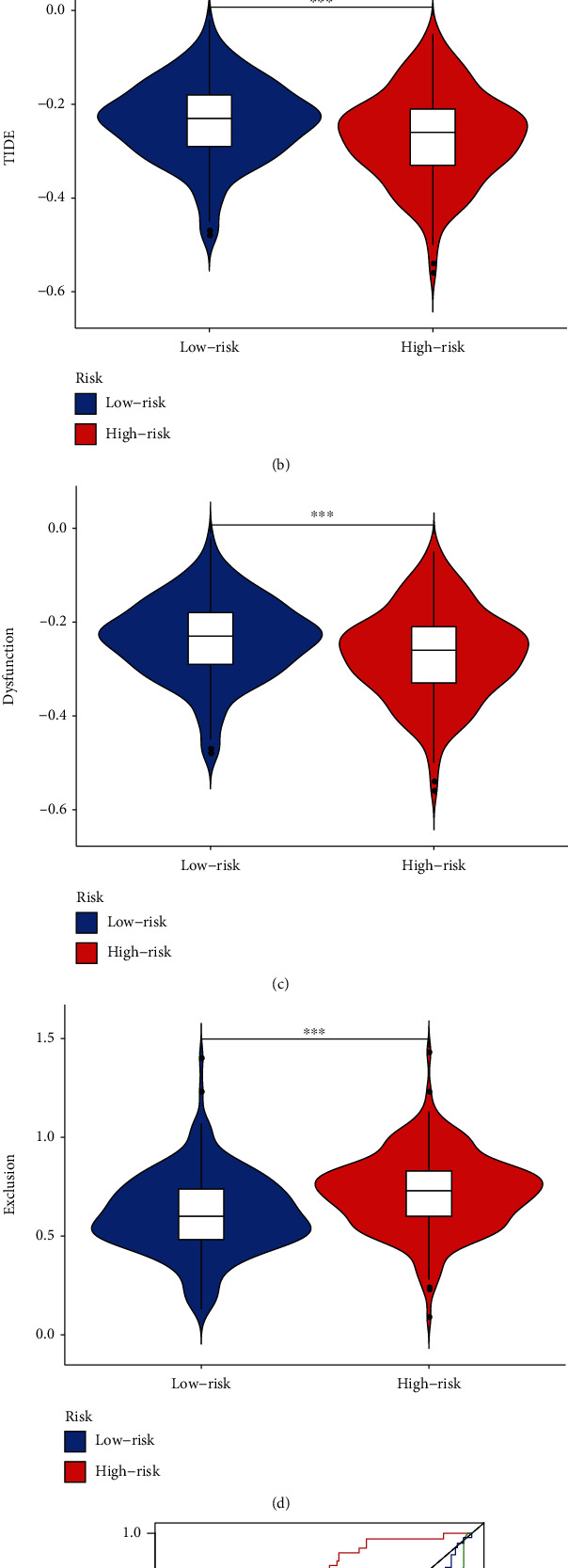
Immune function and TIDE analysis. (a) The expression of immune checkpoints in the high- and low-risk groups. (b) TIDE, (c) dysfunction score, (d) T cell exclusion in the high- and low-risk groups. (e) Time-dependent ROC curves analysis of signature, TIDE, and TIS.

We analyzed the correlation between the signature and the immune cell subtype infiltration, which showed that the signature was positively associated with multiple immune cells including CD8+ T cells, B plasma cells, B memory cells, and B naive cells (Figures 13(a) – 13(g)). Compared with the high-risk group, the abundance of infiltrating CD8+ T cells in the low-risk group was significantly higher. To figure out the relationship between the signature and the expression of immune checkpoint in PCa, we found that the high-risk group was positively correlated with high expression of TIGIT, LAG3, PD-1, and CTLA-4 (Figure 14(a)). The TIDE was applied to evaluate the potential response of ICIs for PCa patients (Figures 14(b) – 14(d)). TIDE value in the high-risk group was significantly lower than that in the low-risk group, demonstrating that the high-risk group deserved a better immunotherapy response and immunotherapy outcome. The time-dependent ROC analysis revealed that the prognostic performance of the signature was significantly higher than that of the newly discovered biomarkers including TIDE and TIS (Figure 14(e)).

### 3.10. Correlation Analysis between the Signature and Drug Treatments

**Figure 15:**
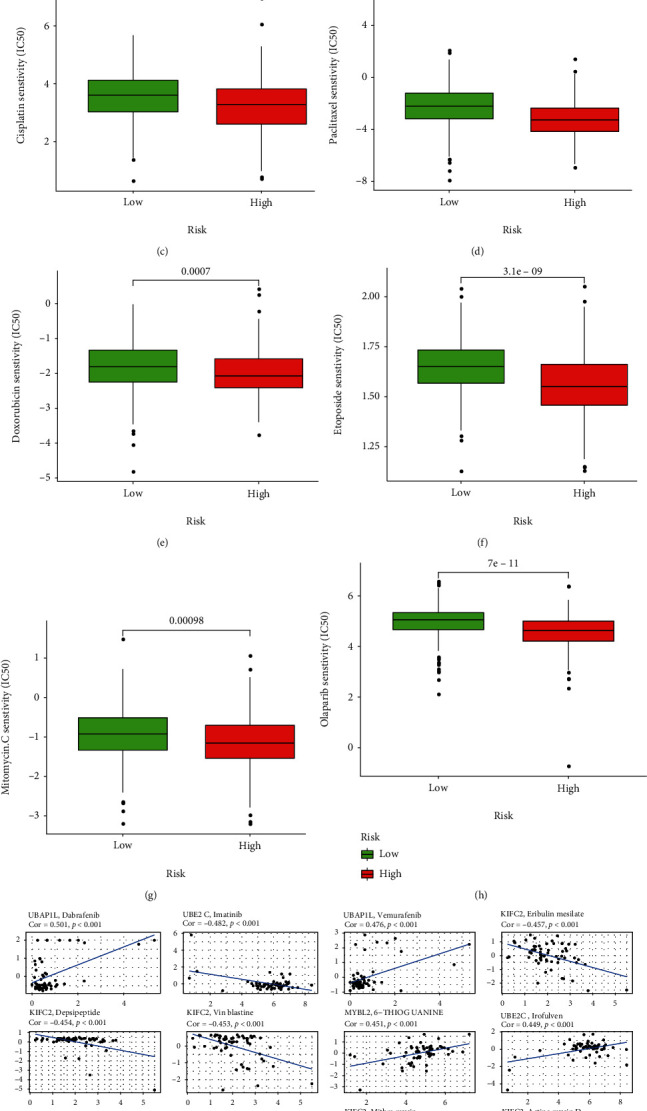
Assessment of the drug sensitivity. The high- and low-risk groups had significant differences in IC50 of drugs such as (a) bicalutamide, (b) docetaxel, (c) cisplatin, (d) paclitaxel, (e) doxorubicin, (f) etoposide, (g) mitomycin C, and (h) olaparib. (i) The relation between multiple drugs and 7 genes.

Endocrine drugs and chemotherapeutic drugs are the conventional options for the nonsurgical treatment of PCa. Therefore, we analyzed the sensitivity of different risk groups to endocrine drugs, which suggested that bicalutamide had a lower IC50 in the low-risk group (Figure 15(a)). Chemotherapy combined with immunotherapy has been shown to have better efficacy than either therapy alone. Our results indicated that patients in the low-risk group were more sensitive to docetaxel. (Figure 15(b)) However, the high-risk group was more sensitive to chemotherapeutic agents such as cisplatin, paclitaxel, doxorubicin, etoposide, and mitomycin C than the low-risk group, implying that patients in the high-risk group were more likely to benefit from these agents (Figures 15(c) – 15(g)). Olaparib, a novel targeted drug, acted to inhibit poly ADP ribose polymerase protein [26]. The high-risk group was more sensitive to olaparib than the low-risk group (Figure 15(h)). Finally, we found that each of the seven genes was also closely related to multiple drugs (Figure 15(i)).

## 4. Discussion

Treatment strategies for PCa have evolved and progressed tremendously over the past decade yet remained unsatisfactory. More than half of patients with high-risk PCa experienced BCR postoperatively [27]. BCR was a significantly poor prognosis for PCa patients and was strongly associated with progression to metastatic castration-resistant prostate cancer (mCRPC) [28]. Accurately predicting the risk of BCR in PCa patients was essential for the clinical management of PCa and the prognosis of patients. Effective management of PCa could be achieved by precisely stratifying patients at low risk of BCR progression from those at high risk of BCR progression. Watchful waiting (active surveillance) and curative therapies of patients at different risks of developing BCR could lead to a better prognosis for the patient population in greater need. However, there was currently no feasible way for risk stratification of PCa patients in clinical practice. Thus, this study focuses on a novel type of programmed cell death pyroptosis that played a complex and important role in tumor development and treatment. Normal cells might be transformed into cancer cells by the inflammatory factors released during the process of pyroptosis [29]. Meanwhile, the interaction between pyroptosis and immune cells in TME affected immune defense and antitumor immune function, which in turn had a significant impact on tumor growth, invasion, and metastasis [30]. Providing a novel and comprehensive insight into the relationship between pyroptosis and TME could lead to better identification of PCa and more precise treatments for the patients. As the first report of pyroptosis-related genes in PCa, this study accurately and effectively classified the risk of PCa patients by constructing a signature, which could predict the BCR and sensitivity to chemotherapy, endocrine therapy, and immunotherapy for PCa patients at different risk groups. Our signature could provide clinicians with new ideas for managing the risk of BCR in PCa patients and guiding clinical treatment strategies.

In this study, first, we determined the expression levels of 33 known pyroptosis-related genes in PCa and normal tissues and identified 22 differentially expressed pyroptosis-related genes related to prognosis. Second, sample classification based on predefined gene expression features was a proven method [31]. In order to verify the prognostic value of pyroptosis-related genes, we found that the expression of pyroptosis-related genes occurred differently in patients divided into two groups, resulting in a completely different prognosis. Patients in cluster 2 had higher expression levels of pyroptosis-related genes and a poorer prognosis. Third, a signature composed of 7 genes through Lasso regression analysis was constructed. The independent and powerful ability of the signature to predict the prognosis of PCa patients was verified in the two independent datasets GSE116918 and GSE21034. Fourth, our signature that was closely associated with various stages of PCa could effectively judge the prognosis of patients in different pathological conditions. There were significant differences between the two risk groups in N stage, T stage, and tumor stage and grade, suggesting that our signature was closely related to the existing clinical characteristics. A total of 5 grading groups from grade 1 to grade 5 were proposed based on the Gleason score [32]. Our results found that our signature was closely related to grade, and that grade increased with increasing risk score, indicating that our signature was strongly associated with the existing scoring systems such as Gleason score. Additionally, we then constructed a nomogram that combined our signature and clinical characteristics to predict the 1-, 2-, and 3-year BCR-free survival rates of PCa patients. Fifth, we compared our signature with nine published signatures constructed for PCa and showed that our signature possesses excellent and accurate prognostic performance superior to the currently established PCa signatures. Overall, our signature had the unexpected predictive ability as well as excellent predictive accuracy to classify PCa patients according to the risk of BCR, which would facilitate clinicians to better treat patients with higher risk.

Chronic inflammation and the associated sustained immune response were thought to contribute to the development and progression of PCa [33]. Pyroptosis was an inflammatory programmed cell death caused by inflammatory caspases and was involved in the inflammatory response to enhance host protective immunity [34]. The tumor microenvironment played a key role in the pathogenesis and disease progression. As the interaction between cancer cells and the tumor microenvironment triggered complex physiological changes that lead to disease severity, cancer metastasis, and resistance to conventional therapies [35]. Q. Wang et al. found that less than 15% pyroptosis of tumor cells could induce the elimination of entire 4T1 tumor grafts in tumor-bearing mice by activating cytotoxic T cells and CD4+ T helper cells in the TME, which was not reproduced in immunodeficient mice [36]. The plasma B cells were considered to be the driving factor of the immune response of PCa, which could improve recurrence-free survival after surgery, and the way that plasma cells participated in the immune system for therapy might be a potential biomarker of the target for therapeutic response to immunotherapy for future prospective evaluation [37]. CD8+ T cells were active antitumor lymphocytes with strong prognostic relevance in many solid tumors [38]. Vicier et al. revealed that low density of CD8+ T cells was influential as an independent poor prognostic marker for BCR and risk of metastatic recurrence in a study of 109 patients with primary PCa [39]. Collectively, it could be seen that the poor prognosis and outcome of PCa were closely related to immune cell infiltration, which was consistent with our results. As we have discovered, the patients in the high-risk group had a significantly shorter time to BCR, while the high-risk group was negatively associated with the immune cells such as CD8+ T cells and plasma B cells. The signature distinguished different groups and thus determined different degrees of immune cell infiltration, leading to different outcomes in PCa. Paying more attention to immune cell infiltration might become a future treatment strategy and further affect the clinical outcome of PCa patients.

One promising PCa treatment method currently under study was immunotherapy, which used the antitumor immune response of the innate immune system to destroy tumorigenesis. ICB therapy targeting CTLA-4, PD-1, and PD-L1 had shown significant therapeutic benefit and become an attractive treatment option for several malignant cancers, such as melanoma, bladder cancer, and lung cancer [40]. It was previously widely believed that PCa did not show a desirable therapeutic response to immunotherapy. However, a small percentage of PCa patients had shown impressive and durable responses to immunotherapy PD-1 inhibition according to the results of KEYNOTE-028 trial [41]. Meanwhile, the immunosuppressive microenvironment of PCa suppressed tumor-specific T cell responses and promoted tumor progression and invasion. A renewed focus on the tumor immune environment was needed to determine prognostic and predictive biomarkers and to guide novel immunotherapies for precise cancer treatment. KEYNOTE-199, the largest ongoing clinical study to date evaluating anti-PD-1 therapy in mCRPC, noted that patients with higher TMB after treatment with pembrolizumab were strongly associated with better prostate-specific antigen (PSA) response and time to PSA progression [42]. Moreover, in the subgroup of patients with mCRPC receiving docetaxel and endocrine therapy, pembrolizumab demonstrated favorable antitumor activity and disease control, which was durable and encouraging [43]. As seen above, a key challenge in managing PCa was clinical heterogeneity, where patients with the same disease may have different outcomes depending on the tumor microenvironment and whether they were treated with a combination of chemotherapy and endocrine therapy, which was difficult to predict with the available biomarkers. In this study, we tried to provide novel insight to explore the immune landscape and immunotherapy in PCa by our signature. We compared the expression of immune checkpoints in the high- and low-risk groups and found that most immune checkpoints such as PD-1, CTLA-4, LAG3, and TIGIT were more expressed in the high-risk group than in the low-risk group. The previous studies reported that increased expression of PD-1 and PD-L1 was associated with more aggressive PCa [44, 45], which was in line with our findings that patients in the high-risk group were more likely to develop BCR and were associated with high-grade and advanced-stage PCa. Meanwhile, patients with high levels of immune checkpoint gene expression were prone to develop immunosuppressive microenvironment to promote tumor immune escape [46], suggesting that PCa patients in the high-risk group were more likely to benefit from immune checkpoint inhibitor therapy. TMB, TIS, and TIDE were newly identified predictors of immunotherapy [16, 47]. In particular, TIDE had been shown to have better performance than other biomarkers or indicators in predicting immunotherapeutic response [48]. We adopted TIDE to assess the potential clinical efficacy of immunotherapy in the high- and low-risk groups. Higher TIDE represents less likely to benefit from immunotherapy, such as PD-1 and CTLA-4 inhibition therapy. Based on our results, patients in the high-risk group with low TIDE were more suitable for immunotherapy. Our signature sheds new light on the effective identification of subgroups of PCa patients who can benefit from immunotherapy. In addition, by comparing the AUC values of our signature with other biomarkers in time-dependent ROC analysis, we observed that our signature had better predictive performance and superiority. Therefore, it was suggested that our signature was not only effective as an efficacy predictor to discriminate PCa patients with greater benefit from immunotherapy but also had higher accuracy and specificity to predict the prognosis than other existing biological indicators. We have proved that our signature could effectively stratify the risk of PCa patients into subgroups that were more suitable for immunotherapy and had the potential as an indicator of immunotherapy response in PCa.

Bicalutamide is a nonsteroidal androgen receptor inhibitor widely used in the endocrine therapy of PCa. A prospective randomized trial demonstrated that the use of bicalutamide significantly reduced the risk of objective disease progression in patients with locally advanced PCa [49]. The sensitivity analysis of bicalutamide in the high- and low-risk groups revealed that the low-risk group had a lower IC50, which meant that patients in the low-risk group had a higher sensitivity for bicalutamide. Chemotherapy is a common treatment for advanced PCa, among which docetaxel is the first choice for chemotherapy in most cases. Combined docetaxel and prednisone was the first-line treatment for mCRPC [50]. Chemotherapy drugs were designed to attack rapidly dividing cells, which include not only cancer cells but also normal cells in the body, and this is where the side effects of chemotherapy arise. The side effects of chemotherapy were determined by the type of drug and the dose and period of taking the drug. Common side effects included hair loss, diarrhea, and infections [51]. However, there was currently no biological indicator for the choice of chemotherapy drugs used in clinical practice. Our results showed that patients in the low-risk group were more sensitive to docetaxel and patients in the high-risk group could benefit more from cisplatin, doxorubicin, etoposide, mitomycin C, and paclitaxel. Subgroups of prostate patients stratified according to the signature had different sensitivities to chemotherapeutic agents. Targeted administration of chemotherapeutic agents based on their sensitivity will not only improve treatment outcomes but also reduce the adverse effects of chemotherapy. In addition, the available clinical trial results indicated that the targeted drug olaparib could bring unexpectedly better results to PCa patients [52]. Our results showed that the high-risk group was more likely to benefit from olaparib. Our signature was a promising and reliable predictor of chemotherapy, endocrine, and targeted therapy in PCa, providing a novel approach to get a better prognosis for patients.

## 5. Conclusion

In short, we have constructed a pyroptosis-related signature that could serve as an independent prognostic factor for PCa. The role of the signature in the immune landscape and treatments was fully elaborated. It was expected to become a robust and promising signature to guide the treatment of PCa.

## Data Availability

All data generated or analyzed during this study are included in this article or are available from the corresponding author on reasonable request.

## Supplementary Materials

Supplementary Materials The sequences of all the primers.
