# Supplementary material for: Characterization of a Pyroptosis-Related Signature for Prognosis Prediction and Immune Microenvironment Infiltration in Prostate Cancer
Source: Comput Math Methods Med. 2022 Apr 27;2022:8233840. doi: 10.1155/2022/8233840 (PMC9066377; doi:10.1155/2022/8233840)
Supplement: Supplementary Materials — The sequences of all the primers. [file 8233840.f1.docx]

**Supplementary Materials**

**The sequences of all the primers**

| **Name** | **Oligonucleotide sequence (5' - 3')** | |
| --- | --- | --- |
|  | **Forward** | **Reverse** |
| **UBAP1L** | GAGCCACAAGCCTACGGTC | CCGCAGTATCAGGGTGTGAC |
| **UBE2C** | GACCTGAGGTATAAGCTCTCGC | CAGGGCAGACCACTTTTCCTT |
| **KIFC2** | AAGGGAAATATCCGTGTGCTG | GTCTAGGCGGAATCGACGATG |
| **MAPK8IP3** | GTGTACCAGGACGACTACTGC | GCACCGAGTCTAGGTTCTCCA |
| **TTLL3** | CCTCTCCGCAGGATGGTTTC | ACAGCTCTCTCCACGTAGATTT |
| **MYBL2** | CCGGAGCAGAGGGATAGCA | CAGTGCGGTTAGGGAAGTGG |
| **MMP11** | CCGCAACCGACAGAAGAGG | ATCGCTCCATACCTTTAGGGC |
| **β-actin** | GCAAAGACCTGTACGCCAACA | TGCATCCTGTCGGCAATG |
